# The effect of hand hygiene frequency on reducing acute respiratory infections in the community: a meta-analysis

**DOI:** 10.1017/S0950268822000516

**Published:** 2022-03-21

**Authors:** Yin Mo, Thi Mui Pham, Cherry Lim, Peter Horby, Andrew J. Stewardson, Stephan Harbarth, Geoffrey M. Scott, Ben S. Cooper

**Affiliations:** 1 Nuffield Department of Medicine, Centre for Tropical Medicine and Global Health, University of Oxford, Oxford OX3 7BN, UK; 2 Mahidol-Oxford Tropical Medicine Research Unit, Faculty of Tropical Medicine, Mahidol University, Bangkok 10400, Thailand; 3 Division of Infectious Diseases, University Medicine Cluster, National University Hospital, Singapore 119074, Singapore; 4 Department of Medicine, National University of Singapore, Singapore 119228, Singapore; 5 Julius Center for Health Sciences and Primary Care of the UMC Utrecht, Utrecht University, Utrecht 3584 CG, The Netherlands; 6 Department of Infectious Diseases, The Alfred Hospital and Central Clinical School, Monash University, Melbourne, Victoria VIC 3004, Australia; 7 Infection Control Program, World Health Organization Collaborating Centre on Patient Safety, Geneva University Hospitals and Faculty of Medicine, Geneva 1205, Switzerland; 8 Clinical Microbiology, University College London Hospitals, London W1T 4EU, UK

**Keywords:** Acute respiratory tract infections, hand hygiene, public health policy

## Abstract

Hand hygiene is a simple, low-cost intervention that may lead to substantial population-level effects in suppressing acute respiratory infection epidemics. However, quantification of the efficacy of hand hygiene on respiratory infection in the community is lacking. We searched PubMed for randomised controlled trials on the effect of hand hygiene for reducing acute respiratory infections in the community published before 11 March 2021. We performed a meta-regression analysis using a Bayesian mixed-effects model. A total of 105 publications were identified, out of which six studies reported hand hygiene frequencies. Four studies were performed in household settings and two were in schools. The average number of handwashing events per day ranged from one to eight in the control arms, and four to 17 in the intervention arms. We estimated that a single hand hygiene event is associated with a 3% (80% credible interval (−1% to 7%)) decrease in the daily probability of an acute respiratory infection. Three of these six studies were potentially at high risk of bias because the primary outcome depended on self-reporting of upper respiratory tract symptoms. Well-designed trials with an emphasis on monitoring hand hygiene adherence are needed to confirm these findings.

## Introduction

Hand hygiene has long been an important component of infection prevention and control policies following the work by Ignaz Philip Semmelweis demonstrating that hand washing could dramatically reduce maternal mortality due to puerperal fever [1]. It is advocated by the World Health Organization (WHO) as a key intervention to reduce the spread of pathogens and prevent infections, including severe acute respiratory syndrome coronavirus 2 (SARS-CoV-2) [2]. National hand hygiene campaigns in hospital settings have seen major successes in reducing healthcare-associated infections caused by multi-drug-resistant organisms such as meticillin-resistant *Staphylococcus aureus* [3]. In the community, hand hygiene interventions have been shown to be effective in reducing both the risk of diarrhoeal disease and acute respiratory infections, though the effect sizes, especially in the latter, are highly variable [4–7]. A recent meta-analysis studying the effect of hand hygiene reported that the associated relative risk reduction was 0.47 (95% confidence interval 0.19–1.12) [8].

Randomised controlled trials which study hand hygiene often attempt to promote hand hygiene through educational messages and providing alcohol handrubs or soaps to the participants allocated to the intervention arms. How successful each trial is in improving hand hygiene behaviour in the intervention arm depends on intervention implementation and participants’ susceptibility to behaviour change. Therefore, trial estimates encapsulate both the effect of hand hygiene behaviour modification and hand hygiene itself. It is important to disentangle these two effects as behaviour change is difficult to achieve and highly context specific. Understanding how the effect of hand hygiene in reducing infection risk scales with hand hygiene frequency is important for assessing the potential contribution of hand hygiene in reducing infection risk and suppressing epidemics, and for motivating public health campaigns. However, the relationship between hand hygiene frequency and change in infection risk has not previously been quantified.

This is especially pertinent in view of the coronavirus disease 2019 (COVID-19) pandemic, where hand hygiene has been re-emphasised as a primary focus in public information campaigns [9, 10]. The lack of specific recommendations on when and how often the public should wash their hands reflects the lack of a quantitative understanding of how different levels of hand hygiene behaviour affect transmission risk.

We addressed this knowledge gap by performing a systematic review and meta-regression analysis considering randomised controlled trials of hand hygiene interventions in community settings which both recorded hand hygiene behaviour and included respiratory tract infection as outcomes.

## Methods

### Systematic review

We searched PubMed for randomised controlled trials on the effect of hand hygiene on reducing acute respiratory infections published before 11 March 2021. The search was performed using ‘hand hygiene’ and ‘respiratory infection’, within the ‘clinical trial’ publication type. The search terms were as follows: (‘*hand hygiene*’[*MeSH Terms*] *OR* (‘*hand*’[*All Fields*] *AND* ‘*hygiene*’[*All Fields*]) *OR* ‘*hand hygiene*’[*All Fields*]) *AND* (‘*respiratory tract infections*’[*MeSH Terms*] *OR* (‘*respiratory*’[*All Fields*] *AND* ‘*tract*’[*All Fields*] *AND* ‘*infections*’[*All Fields*]) *OR* ‘*respiratory tract infections*’[*All Fields*] *OR* (‘*respiratory*’[*All Fields*] *AND* ‘*infection*’[*All Fields*]) *OR* ‘*respiratory infection*’[*All Fields*]) *AND Clinical Trial*[*ptyp*]. In addition, the reference lists from relevant systematic reviews were also searched. Titles and abstracts of the records identified through the initial search were screened for eligibility.

To be eligible for inclusion in the meta-analysis, studies were required to meet all the following criteria:

- randomised controlled trial;
- hand hygiene as the main intervention;
- respiratory tract infection (defined by any combination of respiratory tract symptoms, or with laboratory confirmation of respiratory tract pathogens) as the primary outcome;
- community setting; and
- either direct recording of the number of times study participants washed their hands per day (e.g. by observation or self-report) or indirect recording (e.g. by volume of alcohol hand rub or weight of soap used)

Three authors independently reviewed then verified the data extracted from the shortlisted studies. We contacted the study investigators to confirm and seek missing data not found in the publications. Quality assessment of the studies was performed using the Cochrane risk-of-bias tool for randomised trials (RoB 2 tool) [11]. The PRISMA checklist is available in the Supplementary material.

### Meta-regression

Data from these randomised trials were used in a meta-regression analysis to estimate the change in the daily risk of acquiring a respiratory tract infection per handwash and per hour of face mask worn. Face mask use was included in the analysis because half of the trials had intervention arms where face masks were used alongside hand hygiene promotion.

We used a mixed-effects model, allowing slopes and intercepts to vary between trials. In the models, we assumed that the infection events occur independently, i.e. clustering effects at household level, etc., are ignored. Accounting for such effects was not possible due to lack of information reported in the trials. Had it been possible, the impact would have been increase uncertainty in model estimates. Also, we assumed a linear relationship between the log of the daily infection risk and hand hygiene frequency and hours of mask use. Full details of the meta-regression models are as follows.

Let *p_ij_* be the daily probability for an individual to acquire an acute respiratory tract infection in arm *j* of study *i*, where *i* *=* 1, …, 6 and *j* *=* 1, 2 or 3. Then the model relating this to handwashing and mask use is:
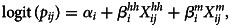
where *X^hh^* is the daily frequency of handwashing, *X^m^* is the daily number of hours of face mask use. Aside from one study in a school setting where handwashing was mandated and adherence was directly observed, handwashing and face mask use were self-reported, i.e. a single value reported per participant. Hence, *X^hh^* and *X^m^* are average values across all participants in each study arm.

The intercepts, *α_i_*, and slopes, *β_i_^hh^* and *β_i_^m^*, are allowed to vary between trials and assumed to be normally distributed.

The number of cases, *Y_ij_*, in arm *j* of study *i* is considered in the two types of studies:

- For studies which reported only one outcome per study participant (which typically had short follow-up periods), the outcomes were modelled with a binomial distribution where the probability of infection over the follow-up period is given by one minus the probability of remaining infection-free over this period:
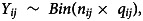

where *n*_*ij*_ is the total number of participants in arm *i* of trial *j* and *q*_*ij*_ is the probability of infection over a follow-up period of *d*_*i*_ days, which is given by 1 minus the probability of remaining free of infection over this period: 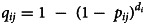.

- For studies which allowed for multiple infections per participant (which typically had long follow-up periods), the outcomes were modelled with a Poisson distribution:
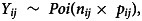

where *n*_*ij*_ is the number of person days at risk and *n*_*ij*_ ×  *p*_*ij*_ is the expected number of infections for the whole follow-up period.

Most studies included in the meta-analysis reported hand hygiene adherence in all randomisation arms (control, hand hygiene, mask), in terms of daily frequency or volumes of soaps/alcohol handrubs used. When volumes or weights of soaps/alcohol handrubs used were reported, we assumed each handwash used 2 ml of alcohol hand rub or 0.35 g of bar soap [12]. In some studies (exclusively in studies which provided alcohol handrubs to participants in the intervention arms), only hand hygiene adherence in the intervention arms was reported. Here we assumed the baseline hand hygiene frequency was four times per day (or six times per day in the sensitivity analysis). We used this baseline value as the hand hygiene frequency in the control arm, and added this baseline value to the reported hand hygiene frequencies in the intervention arms.

When considering hours of mask wearing, we assumed in one study which reported the number of face masks instead of hours of mask use that each mask was worn for an average of 2 h (or 4 h per mask in the sensitivity analysis). We considered relatively short number of hours per mask used because in all studies which used masks as an intervention, participants were given single-use surgical masks. In the arms which did not include mask as an intervention, we assumed the hours of mask worn was zero.

The hand hygiene frequencies and hours of mask worn for each study arm are shown in Table 1 (detailed explanations are included in https://github.com/moyinNUHS/HHCOVID_metaanalysis/). We assessed the sensitivity of the meta-regression results to the above assumptions (Supplementary Table S3). We implemented the above meta-regression model in Stan, and performed all analyses in R version 3.6.2 [13] using the Rstan package [14].

**Table 1.** Details of the six randomised controlled trials included in the meta-analysis

	Country	Year(s) of study	Setting	Randomisation arms	Mean hand hygiene frequencies per day^a^	Only one episode reported per participant	Outcome	Proportion with outcome in HH and mask arm	Proportion with outcome in HH arm	Proportion with outcome in mask arm	Proportion with outcome in control arm	Ref
1	USA	2007–2008	University hall	HH and mask *vs.* mask *vs.* control	10.1 (s.d. 4.1) *vs.* 7.1 (s.d. 1.8) *vs.* 7.4 (s.d. 2.5)	Yes	ILI	31/349 individuals (12 halls)	–	46/392 (13 halls)	51/370 individuals (12 halls)	Aiello *et al*. [15]
							LCI	6/349 individuals (12 halls)		12/392 (13 halls)	16/370 individuals (12 halls)	
2	Thailand	2008–2009	Households (with an index LCI case)	HH and mask *vs.* HH *vs.* control	4.7 (95% CI 4.3–5.0) *vs.* 4.9 (95% CI 4.5–5.3) *vs.* 3.9	Yes	ILI	51/291 individuals (110 households)	50/292 individuals (119 households)	–	26/302 individuals (119 households)	Simmerman *et al*. [16]
							LCI	66/291 individuals (110 households)	66/292 individuals (119 households)		58/302 individuals (119 households)	
3	USA	2006–2008	Households	HH and mask *vs.* HH *vs.* control	5.7 *vs.* 5.5 (HH frequency not reported in control arm)	No	ILI	938/50 676 person-days in 1377 individuals (166 households)	946/48 731 person-days in 946 individuals (169 households)	–	904/46 526 person-days in 904 individuals (174 households)	Larson *et al*. [17]
4	India	2007–2008	Households	HH *vs.* control	16.8 *vs.* 3.2	No	ILI	–	18 432/1 303 281 person-days in 4863 individuals (847 households)	–	20 526/1 288 654 person-days in 4812 individuals (833 households)	Nicholson *et al*. [18]
5	Germany	2009–2011	Households (with an index LCI case)	HH and mask *vs.* mask *vs.* control	4 *vs.* 1	Yes	ILI	6/67 individuals (28 households)	–	6/69 individuals (26 households)	14/82 individuals (30 households)	Suess *et al*. [19]
							LCI	10/67 individuals (28 households)		6/69 individuals (26 households)	19/82 individuals (30 households)	
6	Thailand	2009–2010	School	1-hourly HH *vs.* 2-hourly HH *vs.* control	6 *vs.* 3 *vs.* 1	No	Absenteeism due to ILI	–	1-hourly HH: 438/37 716 person-days in 452 individuals (21 classes) 2-hourly HH: 501/37 716 person-days in 452 individuals (21 classes)	–	644/45 360 person-days in 540 individuals (26 classes)	Pandejpong *et al*. [20]

HH, hand hygiene; ILI, influenza-like illness; LCI, laboratory-confirmed influenza.

a Standard deviations, confidence intervals are included when reported.

## Results

### Randomised trials included in the meta-analysis

**Fig. 1.**
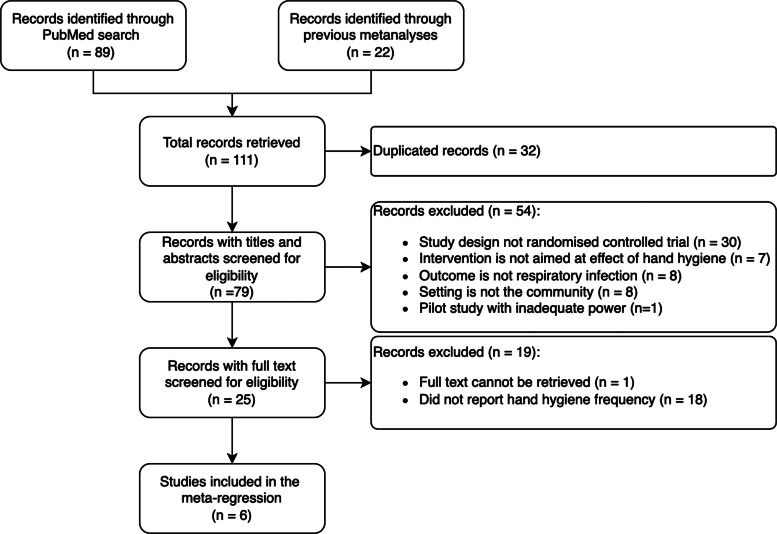
PRISMA flow diagram. The flow diagram shows the number of studies identified, reviewed, included and excluded in the meta-analysis.

A total of 111 publications were identified through PubMed searches and previous meta-analyses on the effect of hand hygiene on respiratory tract infections in the community [5, 6, 21]. The PRISMA diagram is shown in Figure 1.

Twenty-five trials were reviewed in detail (Supplementary Table S1); all were performed prior to the COVID-19 pandemic, and 17 (68%) monitored hand hygiene adherence during the conduct of the trial. The most common method used was self-reported handwashing frequency (8/17, 47%). Direct observation was done in three studies (3/17, 18%) performed in schools or childcare settings.

Six studies reported hand hygiene frequencies in at least one of the study arms (Table 1). Overall, the average number of handwashing events per day was found to range from one to eight in the control arms, and four to 17 in the intervention arms. Four were performed in household settings, one in a primary school and one in a university setting. All of the studies included children or young adults, and most were carried out in high (3) or upper-middle (2) income countries with one in low-income families in a lower-middle income country. Four studies were carried out either exclusively during influenza seasons when performed in countries with well-defined influenza seasons or had extended periods of enrolment or follow-up which included an influenza season. The other two studies were carried out in Thailand where there is no clearly defined influenza season. In the four studies which performed laboratory confirmation of the viral pathogen presumed to be causing the respiratory symptoms, only one tested for respiratory viruses other than influenza.

Four out of the six studies included face mask use as part of the intervention bundle together with hand hygiene to prevent acute respiratory illness. In two, face mask use was studied in an intervention arm on its own. In all the studies involving masks, standard surgical masks were issued to the participants. The self-reported number of hours for which the participants wore face masks per day ranged from 2 to 5 h.

### Results of the meta-regression

**Fig. 2.**
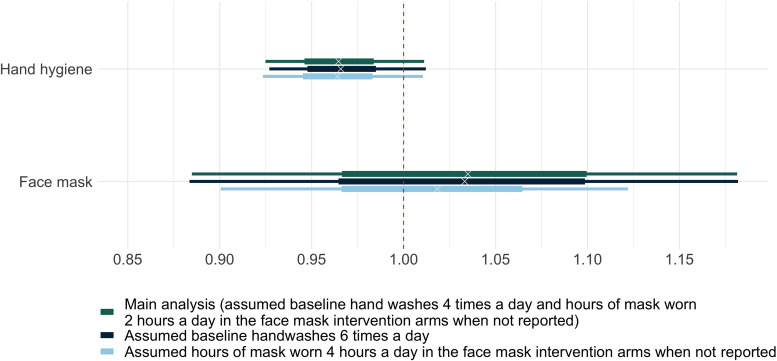
Relative risks of acquiring respiratory tract infection per day with one hand wash or 1 h of face mask worn. The horizontal bars illustrate the posterior distributions of the relative risks of acquiring respiratory tract infection per day derived with the Bayesian meta-regression model. The wide bars represent 50% credible intervals. The narrow bars represent 80% credible intervals.

The meta-regression analysis estimated a relative risk for the daily probability of acquiring a respiratory tract infection with one additional handwash of 0.97 (80% credible interval 0.92–1.01). This corresponds to a point estimate of a 3% reduction in daily risk of acquiring a respiratory tract infection per hand wash, though with a high degree of uncertainty. The corresponding figure for an hour of face mask use was 1.03 (80% credible interval 0.88–1.18), indicating no evidence that use of face masks reduced infection risk, though compatible with important effects in either direction. Sensitivity analyses showed similar estimates when we varied assumptions about the baseline hand hygiene frequency and hours of mask use (Supplementary Table S3 and Fig. 2).

The 3% reduction in daily risk could be extrapolated to a 15% reduction in daily risk of infection with five additional hand hygiene events per day, and a 28% reduction with 10 (assuming no saturation of hand hygiene effects at these frequencies). This estimate comes with considerable uncertainty: there is a 50% probability that the reduction in the daily infection risk associated with a single hand hygiene event lies between 2% and 5% (the 50% credible interval). The 80% credible interval (−1% to 7%) does not rule out the theoretical possibility that hand hygiene increases infection rates. However, the totality of the evidence (including trials not reporting hand hygiene frequency) suggests that this is unlikely.

Out of the six studies included in the meta-analysis, three were assessed to have a high risk of bias, and for two there were some concerns; one was assessed as having a low risk of bias (Supplementary Table S2). The main source of bias identified across the studies was subjectivity in study outcomes; the latter were considered prone to bias because the participants either self-reported upper respiratory tract symptoms, or underwent laboratory testing for upper respiratory tract infections based on self-reported symptoms.

## Discussion

From our analysis, we found that a single hand hygiene event (i.e. handwash with soap and water or application of alcohol hand rub) was associated with a reduction in the daily risk of infection of just over 3%. While there was substantial uncertainty associated with this estimate and much larger benefits or modest harms cannot definitively be ruled out, the findings nonetheless suggest that small improvements in hand hygiene are likely to be associated with small, but potentially important, reductions in infection risk.

The result also provides insight into the modest and heterogeneous effect of hand hygiene reported by controlled trials aiming to reduce respiratory tract infections in the community [5, 6, 21]. There are three major meta-analyses on the effect of hand hygiene in reducing acute respiratory tract infections in the community. Aiello *et al*. found that the overall proportion of respiratory illness prevented by all hand hygiene interventions combined was 21% (95% confidence interval 5–34%) [7]. Warren-Gash *et al*. reported high-quality evidence of a substantial reduction of respiratory infection in childcare settings in low-income countries (rate ratio 0.50, 95% confidence interval 0.38–0.66), but this was not the case in households with an index infected case (rate ratio RR 1.15, 95% confidence interval 0.57–2.32) [5]. Lastly, Wong *et al*. reported a relative risk reduction of 18% (95% confidence interval −2% to 36%) in a pooled analysis focusing on the outcome of laboratory-confirmed influenza [6].

It is unclear to what extent the variability in these estimates is explained by the success in achieving substantial changes in hand hygiene behaviour amongst the study participants in these trials. A common observation in these studies is poor adherence to assigned hand hygiene interventions, possibly due to the difficulties in motivating and sustaining behaviour change. External factors such as seasonal respiratory viral epidemics may also influence hand hygiene behaviour in the whole study population. Hence these trials, which generally report intention-to-treat estimates, evaluate the effect of assigning hand hygiene interventions instead of hand hygiene itself. An additional complication is that the trials used very different interventions, and in many cases combined hand hygiene education with the promotion of the use of face masks. This makes direct comparisons of the trial outcomes difficult.

**Fig. 3.**
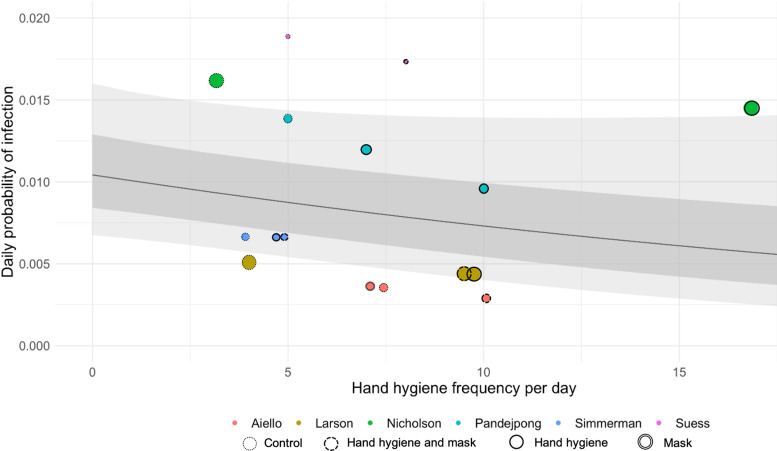
Daily risks of respiratory tract infection given hand hygiene frequencies reported by the six randomised controlled trials included in the meta-analysis. Daily probability of respiratory tract infection (*y*-axis) is shown against hand hygiene frequencies (*x*-axis) reported by each trial. Each colour represents one trial. Each bubble represents a single arm in one trial, where the diameter of the bubble corresponds to strength of evidence that probability of infection takes a particular value (calculated by 1/80% credible interval). The analysis allowed the relationship between hand hygiene frequency and probability of infection to vary across trials; the black line corresponds to the mean relationship between the two considering all included trials. The light grey shaded areas are the associated 80% and 50% credible intervals. The different types of borders around the bubbles indicate the interventions in each trial.

If hand hygiene plays a direct causal role in decreasing infection risk, then there must be a dose–response relationship between hand hygiene frequency and decreased infection risk. However, the parameter values derived from the meta-regression suggest that the risk declines only gradually with increasing hand hygiene frequency. This rate of decline may vary according to study setting (Fig. 3). Most of these trials report only small improvements in handwashing frequency and are therefore unlikely to detect reductions in infection risk. This is further compounded by factors which might dilute the effect of hand hygiene interventions administered in the trials such as poor adherence and misspecification of cases.

It is self-evident that any benefits of increasing hand hygiene frequency must plateau (because reductions of infection risk above 100% are not possible) and an understanding of how incremental benefits of increasing the hand hygiene frequency change with baseline hand hygiene frequency would be useful for informing public health interventions. While the data are too sparse and estimates too uncertain to reliably answer this question here, there are some reasons for thinking that, at least for influenza, worthwhile benefits of improving hand hygiene may continue to accrue even at high hand hygiene frequencies. An example of a study which achieved hand hygiene frequencies much higher than typically observed was performed by Pandejpong *et al*. [20] School-aged students were randomised to wash their hands hourly, two-hourly or before lunch only (control arm) and the study team ensured adherence by sitting in during lessons and giving reminders at scheduled times. Similar reductions in influenza-like-illness infection risk were found when comparing two-hourly to hourly hand hygiene as was seen when comparing hand hygiene before lunch to hourly hand hygiene alone. This study provides some evidence that the benefits of hand hygiene have not maximised even when it is performed at hourly intervals.

A crucial, but often overlooked, determinant of the relationship between hand hygiene frequency and risk of infection is persistence of infectious virus on hands. If virus persists on unwashed hands for long periods, then even relatively infrequent hand hygiene could substantially reduce transmission. Conversely, if the virus survives on hands for only a short time then the interval between hands becoming contaminated and an infectious dose being transferred to the mucosa will also tend to be short [22]. In this case, hand hygiene will need to occur shortly after contamination events to have a good chance of preventing transmission. The half-life of viable influenza A on human fingertips has been reported to be only 5 min for a 2 μl drop of viral suspension mixed with respiratory secretions [23]. With such a rapid decline, one might expect a majority of indirectly transmitted infection events to occur fairly quickly after hand contamination. The high observed frequency of face-touching also supports this recommendation, as this could also lead to short time intervals between hand contamination and infection. Interventions should therefore focus not only on increasing the frequency of hand hygiene, but also on improving its timeliness.

Our findings carry further implications for public health policies for acute viral respiratory epidemics. While the usual key message in hand hygiene campaigns is ‘wash hands often’, the frequency required to reduce infection risk is not specified. From the randomised trials included in our meta-analysis, the baseline hand wash frequency is as low as one to four times a day in the community. Even in outbreak settings, such as the 2015 Korean Middle East Respiratory Syndrome and 2009 H1N1 epidemics, the self-reported number of hand washes with soap or alcohol hand rub was about two to four times a day [24, 25]. A review of hand hygiene behaviour during the SARS outbreak in 2003 showed that though most people in affected areas reported an increase in hand washing frequency during the outbreak, fewer than half washed hands more than 10 times a day and only about two-thirds did so after touching surfaces likely to be contaminated [26]. Even if transmission mediated by fomites is not a dominant route for a particular emerging viral epidemic (as seems to be the case for SARS-CoV-2) [27], even small reductions in the reproduction number can have large effects when the reproduction number is close to one [28] and public health messages on hand hygiene should stress that hand washing should be done as often as practicable and as soon as possible after coming in contact with high-touch areas in shared spaces.

There are important limitations in this analysis. Firstly, the number of hand hygiene randomised trials that reported the participants’ adherence to their allocated intervention was limited. We were only able to include six studies in the meta-analysis, which led to wide credible intervals and potential overfitting. Future trials should consider monitoring adherence as a process measure to assess if hand hygiene behaviour modification was effective through the trial intervention, and to allow inference on actual efficacy of hand hygiene itself. Secondly, we were not able to account for the clustering effect in each study. This clustering effect is likely to increase uncertainty and may alter the estimates. Thirdly, the studies mainly assessed influenza (influenza-like illness and laboratory-confirmed influenza) as the outcome. Because respiratory viruses have different survival times on fomites and human skin and differences in the relative importance of different transmission routes, the efficacy of hand hygiene to curb transmission is expected to vary by pathogen. Another caveat is that the proportion of influenza-like illness due to influenza may vary over space and time and this may affect generalisability of our estimates. In addition, a number of the studies were deemed to be at high risk of bias as they depended on participants’ initiative to report symptoms as primary outcomes. Fourthly, we made a number of assumptions on hand hygiene frequencies and hours of mask use which may deviate from what actually took place during the trials. However, varying these assumptions in sensitivity analyses produced similar results (Supplementary Table S3). Including these variables which reflect adherence to allocated interventions also added additional uncertainties compared to a conventional pairwise meta-analysis. Lastly, as we did not specifically search for randomised trials which studied the effect of mask wearing, and instead focused on hand hygiene trials with masks as a supplementary intervention, we are not able to make conclusions on the effect of masks.

Hand hygiene is simple, low-cost, minimally disruptive and, when widely adopted, may lead to substantial population-level effects in suppressing acute respiratory infection epidemics. Conclusions from our analysis support hand hygiene as an effective infection prevention and control measure in the community setting for acute respiratory epidemics. Mobilising the general public for better hand hygiene practices and improving access to clean water, soap and alcohol hand rub should be emphasised as part of the global response to the ongoing COVID-19 pandemic. Future hand hygiene trials should, where possible, monitor hand washing frequencies.

## Data Availability

The extracted data from the randomised trials included in the meta-analysis and analysis code can be found at https://github.com/moyinNUHS/HHCOVID_metaanalysis. Data are available under the terms of the Creative Commons Zero ‘No rights reserved’ data waiver (CC0 1.0 Public domain dedication).

## Supplementary material

For supplementary material accompanying this paper visit http://doi.org/10.1017/S0950268822000516. click here to view supplementary material
