# Supplementary material for: The effect of hand hygiene frequency on reducing acute respiratory infections in the community: a meta-analysis
Source: Epidemiol Infect. 2022 Mar 21;150:e79. doi: 10.1017/S0950268822000516 (PMC9044525; doi:10.1017/S0950268822000516)

# **Supplementary material**

**Table S1.** Systematic review on the effect of hand hygiene or hand hygiene and face mask on respiratory tract infections.

| **No** | **Country** | **Setting** | **Adherence** | **Outcome** | **Hand hygiene** | **Hand hygiene/mask** | **Ref** |
| --- | --- | --- | --- | --- | --- | --- | --- |
|  |  |  |  |  | **RR (95% CI)** | |  |
| 1 | Bangladesh | Primary school | Not monitored | URTI symptoms | 0.82 (0.77-0.88)^1^ |  | [1] |
| 2 | Spain | Childcare | Not monitored | URTI symptoms | 0.69 (0.57-0.84)^1^ |  | [2] |
| 3 | Saudi Arabia | Primary school | Not monitored | Absenteesm from  URTI symptoms | 0.51 (0.32-0.82) |  | [3] |
| 4 | Finland | Office | Not monitored | URTI symptoms | 0.84 (0.66-1.03)^2^ |  | [4] |
| 5 | Spain | Primary school | Not monitored | Absenteesm from  URTI symptoms | 0.62 (0.55-0.70) |  | [2] |
| 6 | Bangladesh | Household | Weighing of soap used in the intervention group | LCI | 1.24 (0.93-1.65) |  | [5] |
|  |  |  |  | URTI symptoms | 1.49 (1.05-2.10) |  |  |
| 7 | UK | Household | Not monitored | URTI symptoms | 0·86 (0·83–0·89) |  | [6] |
| 8 | Netherlands | Childcare | Self-reported hand washing frequency | URTI symptoms | 1.01 (0.79-1.29) |  | [7] |
| 9 | New Zealand | Primary school | Volume of alcohol handrub in intervention group | Absenteesm from  URTI symptoms | 1.05 (0.92-1.20) |  | [8] |
| 10 | India | Household | Number of soap wrappers | URTI symptoms | 0.86 (0.77, 0.94) |  | [9] |
| 11 | Germany | Household | Self-reported hand hygiene frequency | LCI |  | 0.49 (0.2-1.6) | [10] |
|  |  |  |  | URTI symptoms |  | 0.26 (0.06-1.17) |  |
| 12 | Thailand | Childcare | Direct observation | Absenteesm from  URTI symptoms | 0.65 (0.36-1.20) for  HW every hour | 0.96 (0.56-1.65) for HW every 2 hours | [11] |
| 13 | USA | University hall | Self-reported hand hygiene frequency | LCI |  | 0.92 (0.26-1.24) | [12] |
|  |  |  |  | URTI symptoms |  | 0.78 (0.57-1.08) |  |
| 14 | Thailand | Households | Self-reported hand hygiene | LCI | 1.18 (0.86-1.61) |  | [13] |
|  |  |  |  | frequency | URTI symptoms | 1.99 (1.27-3.11) |  |
| 15 | USA | Primary school | Direct observation and volume of alcohol handrub | LCI | 0.81 (0.54-1.23) |  | [14] |
|  |  |  |  | Absenteesm from  URTI symptoms | 0·86 (0·60–1·22) |  |  |
| 16 | Egypt | Primary school | Direct observation | LCI | 0·50 (0·38–0·66) |  | [15] |
|  |  |  |  | URTI symptoms | 0·62 (0·49–0·78) |  |  |
| 17 | USA | University hall | Self-reported hand hygiene  frequency | LCI |  | 1.0 (0.2-6.0) | [16] |
|  |  |  |  | URTI symptoms |  | 0.87 (0.73–1.02) |  |
| 18 | Germany | Office | Self-reported hand hygiene frequency | URTI symptoms | 0.62 (0.45-0.87) |  | [17] |
| 19 | USA | Household | Home visits every two months to record use of alcohol handrub | LCI | 1·15 (0·57–2·32) |  | [18] |
|  |  |  |  | URTI symptoms | 0·91 (0·69–1·20) |  |  |
| 20 | Hong Kong | Household | Self-reported hand hygiene  frequency | LCI | 0.54 (0.29-1.00) |  | [19] |
|  |  |  |  | URTI symptoms | 0.70 (0.31-1.58) |  |  |
| 21 | USA | Household | Volume of alcohol handrub | URTI symptoms | 0.97 (0.72–1.30) |  | [20] |
| 22 | Pakistan | Household | Bars of soaps purchased (HW not monitored) | URTI symptoms/ pneumonia | 0·49 (0·35–0·63) |  | [21] |
| 23 | USA | Primary school | Not monitored | Absenteesm from  URTI symptoms | 0.67 (0.51-0.88) |  | [22] |
| 24 | Australia | Childcare | Self-reported hand hygiene frequency | URTI symptoms | 0.93 (0.86-0.99) |  | [23] |
| 25 | Canada | Childcare | Measured coliforms on hands | URTI symptoms | 0.86 (0.70-1.06) |  | [24] |

Abbreviations: LCI - Laboratory-confirmed influenza; URTI - Upper respiratory tract infections; HW - handwash

^1^ the estimate is for soap and water group, 0.91 (0.75-1.10) for alcohol handrub group

^2^ the estimate is for soap and water group, 0.96 (0.76-1.20) for alcohol handrub group

**Table S2**. Quality assessment of the randomised controlled trials included in the meta-analysis.

| Reference | Effect of interest | Study outcomes | Overall risk of bias |
| --- | --- | --- | --- |
| Aiello 2012 [12] | ITT | LCI amongst self-reported ILI cases (primary) | High risk (Subjectivity in study outcome: throat swabs for LCI is only tested among those with self-reported URI symptoms) |
| Simmerman 2011 [13] | ITT | LCI (primary),  ILI (secondary) | Low risk (Introduction of national hand-hygiene education for the H1N1 epidemic during the trial; subjectivity in secondary study outcome: self-reported URI symptoms) |
| Larson 2010 [18] | ITT | LCI (primary),  ILI (secondary) | Some concerns (Protocol of the study was not available; difference in baseline characteristics between the randomised groups; subjectivity in study outcome: throat swabs for LCI is only tested based on self-reported outcomes) |
| Nicholson 2014 [9] | ITT | Acute respiratory illness (primary) | High risk (Difference in baseline characteristics between the randomised groups; allocation sequence was performed using coin toss and information on who tossed the coin was not provided; acute respiratory symptoms were self-reported and cannot rule out the possibilities of multiple eligible outcome measures within this outcome domain) |
| Suess 2012 [10] | ITT | LCI (primary),  ILI (secondary) | Some concerns (Difference in baseline characteristics between the randomised groups; subjectivity in secondary study outcome:  self-reported URI symptoms) |
| Pandejpong 2012 [11] | ITT | Absenteeism due to  self-reported, physician confirmed ILI (primary) | High risk (Protocol of the study was not available; subjectivity in study outcome: absenteeism which may not reflect true upper respiratory tract infections; unable to rule out that the assessment of the outcome was influenced by the knowledge of intervention received as study participant could not be blinded; subjectivity in primary study outcome: self-reported URI symptoms which may not represent upper respiratory tract infection) |

ITT: Intention-to-treat; URI: upper respiratory tract infection; ILI: Influenza-like illness; LCI: Laboratory-confirmed influenza

References for Tables S1 and S2:

1. **Biswas D, *et al.*** Effectiveness of a behavior change intervention with hand sanitizer use and respiratory hygiene in reducing laboratory-confirmed influenza among schoolchildren in Bangladesh: A cluster randomized controlled trial. *American Journal of Tropical Medicine and Hygiene* American Society of Tropical Medicine and Hygiene, 2019; **101**: 1446–1455.

2. **Azor-Martinez E, *et al.*** Hand Hygiene Program Decreases School Absenteeism Due to Upper Respiratory Infections. *Journal of School Health* Blackwell Publishing Ltd, 2016; **86**: 873–881.

3. **Alzaher AA, *et al.*** The importance of hand hygiene education on primary schoolgirls’ absence due to upper respiratory infections in Saudi Arabia a cluster randomized controlled trial. *Saudi Medical Journal* Saudi Arabian Armed Forces Hospital, 2018; **39**: 1044–1049.

4. **Hovi T, Ollgren J, Savolainen-Kopra C**. Intensified hand-hygiene campaign including soap-and-water wash may prevent acute infections in office workers, as shown by a recognized-exposure -adjusted analysis of a randomized trial. *BMC Infectious Diseases* BioMed Central Ltd., 2017; **17**: 47.

5. **Ram PK, *et al.*** Impact of intensive handwashing promotion on secondary household influenza-like illness in rural Bangladesh: Findings from a randomized controlled trial. *PLoS ONE* Public Library of Science, 2015; **10**: 125200.

6. **Little P, *et al.*** An internet-delivered handwashing intervention to modify influenza-like illness and respiratory infection transmission (PRIMIT): A primary care randomised trial. *The Lancet* Lancet Publishing Group, 2015; **386**: 1631–1639.

7. **Zomer TP, *et al.*** A hand hygiene intervention to reduce infections in child daycare: A randomized controlled trial. *Epidemiology and Infection* Cambridge University Press, 2015; **143**: 2494–2502.

8. **Priest P, *et al.*** Hand sanitiser provision for reducing illness absences in primary school children: A cluster randomised trial. *PLoS Medicine* Public Library of Science, 2015; **11**Published online: 2015.doi:10.1371/journal.pmed.1001700.

9. **Nicholson JA, *et al.*** An investigation of the effects of a hand washing intervention on health outcomes and school absence using a randomised trial in Indian urban communities. *Tropical Medicine and International Health* Trop Med Int Health, 2014; **19**: 284–292.

10. **Suess T, *et al.*** The role of facemasks and hand hygiene in the prevention of influenza transmission in households: Results from a cluster randomised trial; Berlin, Germany, 2009-2011. *BMC Infectious Diseases* BMC Infect Dis, 2012; **12**Published online: 26 January 2012.doi:10.1186/1471-2334-12-26.

11. **Pandejpong D, *et al.*** Appropriate time-interval application of alcohol hand gel on reducing influenza-like illness among preschool children: A randomized, controlled trial. *American Journal of Infection Control* Am J Infect Control, 2012; **40**: 507–511.

12. **Aiello AE, *et al.*** Facemasks, hand hygiene, and influenza among young adults: A randomized intervention trial. *PLoS ONE* PLoS One, 2012; **7**Published online: 25 January 2012.doi:10.1371/journal.pone.0029744.

13. **Simmerman JM, *et al.*** Findings from a household randomized controlled trial of hand washing and face masks to reduce influenza transmission in Bangkok, Thailand. *Influenza and other Respiratory Viruses* Blackwell Publishing Ltd, 2011; **5**: 256–267.

14. **Stebbins S, *et al.*** Reduction in the incidence of influenza A but not influenza B associated with use of hand sanitizer and cough hygiene in schools: A randomized controlled trial. *Pediatric Infectious Disease Journal* Lippincott Williams and Wilkins, 2011; **30**: 921–926.

15. **Talaat M, *et al.*** Effects of hand hygiene campaigns on incidence of laboratory-confirmed influenza and absenteeism in schoolchildren, Cairo, Egypt. *Emerging Infectious Diseases* Centers for Disease Control and Prevention, 2011; **17**: 619–625.

16. **Aiello AE, *et al.*** Effect of hand hygiene on infectious disease risk in the community setting: A meta-analysis. *American Journal of Public Health* American Public Health Association, 2008; **98**: 1372–1381.

17. **Hübner NO, *et al.*** Effectiveness of alcohol-based hand disinfectants in a public administration: Impact on health and work performance related to acute respiratory symptoms and diarrhoea. *BMC Infectious Diseases* BioMed Central, 2010; **10**: 250.

18. **Larson EL, *et al.*** Impact of non-pharmaceutical interventions on URIs and influenza in crowded, urban households. *Public Health Reports* Association of Schools of Public Health, 2010; **125**: 178–191.

19. **Cowling BJ, *et al.*** Facemasks and hand hygiene to prevent influenza transmission in households: A cluster randomized trial. *Annals of Internal Medicine* American College of Physicians, 2009; **151**: 437–446.

20. **Sandora TJ, Shih MC, Goldmann DA**. Reducing absenteeism from gastrointestinal and respiratory illness in elementary school students: A randomized, controlled trial of an infection-control intervention. *Pediatrics* 2008; **121**Published online: June 2008.doi:10.1542/peds.2007-2597.

21. **Luby SP, *et al.*** Effect of handwashing on child health: A randomised controlled trial. *Lancet* Elsevier B.V., 2005; **366**: 225–233.

22. **White CG, *et al.*** Reduction of illness absenteeism in elementary schools using an alcohol-free instant hand sanitizer - PubMed. *Journal of School Nursing*  2001; **17**: 258–265.

23. **Roberts L, *et al.*** Effect of infection control measures on the frequency of upper respiratory infection in child care: A randomized, controlled trial. *Pediatrics* American Academy of Pediatrics, 2000; **105**: 738–742.

24. **Carabin H, *et al.*** Effectiveness of a training program in reducing infections in toddlers attending day care centers - PubMed. *Epidemiology* 1999; **10**: 219–227.

**Table S3.** Sensitivity analyses

Different assumptions on the baseline hand hygiene frequencies and hours of mask use were used in the sensitivity analyses.

|  | Assumptions | Hand hygiene | Face mask |
| --- | --- | --- | --- |
|  |  | RR (80% CrI) | |
| Main model | Baseline hand hygiene frequency is 4, number of hours of mask-use in mask groups (if not reported) is 2 | 0.97 (0.92 - 1.01) | 1.03 (0.88 - 1.18) |
| Sensitivity analysis 1 | Baseline hand hygiene frequency is 6, number of hours of mask-use in mask groups (if not reported) is 2 | 0.97 (0.93 - 1.01) | 1.03 (0.88 - 1.18) |
| Sensitivity analysis 2 | Baseline hand hygiene frequency is 4, number of hours of mask-use in mask groups (if not reported) is 4 | 0.96 (0.92 - 1.01) | 1.02 (0.90 - 1.12) |
| Sensitivity analysis 3 | Different priors using N(0, 5) | 0.96 (0.92- 1.00) | 1.02 (0.90 - 1.12) |

Abbreviations: RR: Relative risk; CrI: Credible interval

## **Model assessment**

Prior distributions were selected to be weakly informative normal distributions. We assessed the models using measures of Markov chain convergence including effective sample sizes and *R*^ˆ^ which indicate if the chains had run for long enough and had mixed well. The split *R*^ˆ^ statistic was proposed in Gelman and Rubin (1992)*.*

Figure S1. Plots of iterations vs. sampled values for model parameters in the MCMC chains. The four different chains are plotted using different colours. In the main analysis model the *R*^ˆ^ values were about 1 and the minimum effective sample size was 20,000 across all parameters.


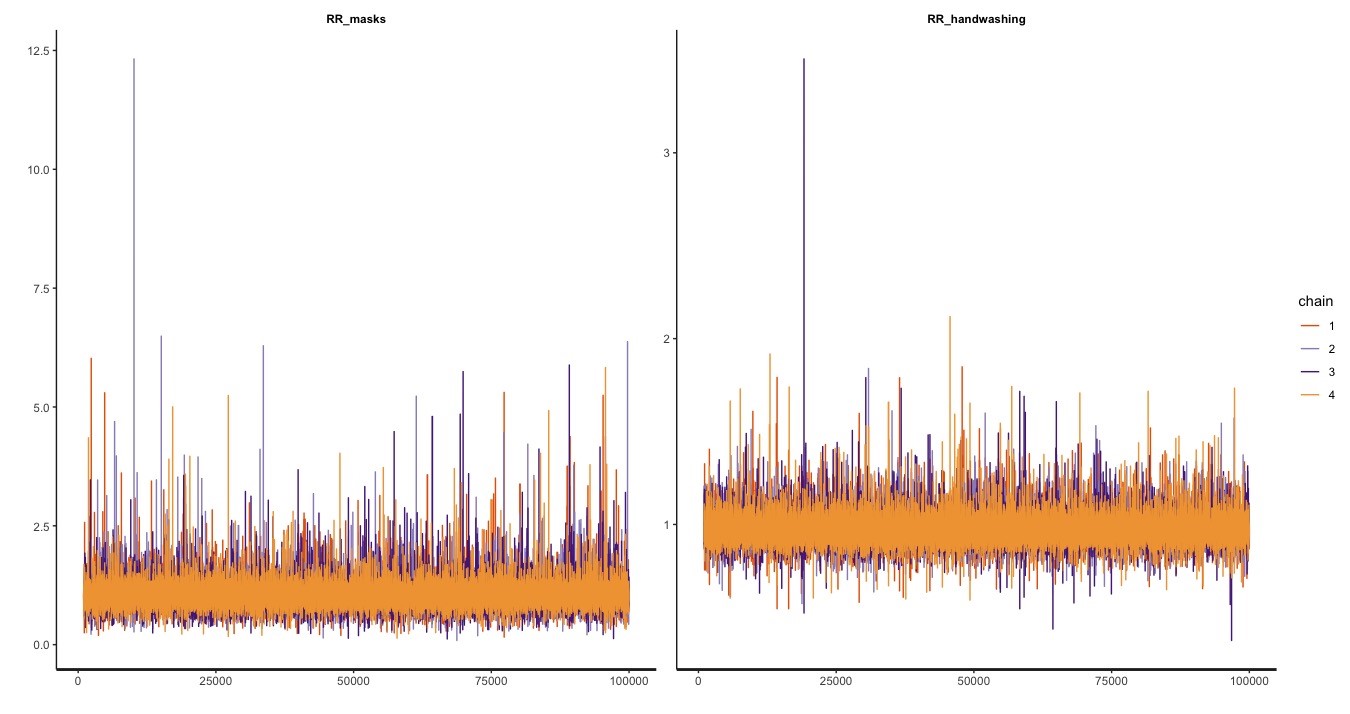

Supplement: Supplementary file 1 [file S0950268822000516sup001.docx]
